# Cardiac and renal upregulation of Nox2 and NF‐*κ*B and repression of Nox4 and Nrf2 in season‐ and diabetes‐mediated models of vascular oxidative stress in guinea‐pig and rat

**DOI:** 10.14814/phy2.13474

**Published:** 2017-10-30

**Authors:** Anna Gajos‐Draus, Monika Duda, Andrzej Beręsewicz

**Affiliations:** ^1^ Department of Clinical Physiology Postgraduate Medical School Warsaw Poland

**Keywords:** Diabetes mellitus, NADPH oxidase homologues, NF‐*κ*B, Nrf2, oxidative stress, seasonality

## Abstract

The superoxide‐forming NADPH oxidase homologues, Nox1, Nox2, and Nox5, seem to mediate the pro‐atherosclerotic vascular phenotype. The hydrogen peroxide‐forming Nox4 afforded vascular protection, likely via NF‐E2‐related factor‐2 (Nrf2) activation and/or Nox2 downregulation in transgenic mice. We hypothesized that oxidative stress in the intact vasculature involves, aside from the upregulation of the superoxide‐forming Noxs, the downregulation of the Nox4/Nrf2 pathway. Guinea‐pigs and rats were studied either in winter or in summer, and the streptozotocin diabetic rats in winter. Plasma nitrite, and superoxide production by isolated hearts were measured, while frozen tissues served in biochemical analyses. Summer in both species and diabetes in rats downregulated myocardial Nox4 while reciprocally upregulating Nox2 and Nox5 in guinea‐pigs, and Nox2 in rats. Simultaneously, myocardial Nrf2 activity and the expression of the Nrf2‐directed heme oxygenase‐1 and endothelial NO synthase were reduced while activity of the nuclear factor *κ*B (NF‐*κ*B) and the expression of NF‐*κ*B‐directed inducible NO synthase and the vascular cell adhesion molecule‐1 were increased. Cardiac superoxide production was increased while plasma nitrite was decreased reciprocally. Analogous disregulation of Noxs, Nrf2, and NF‐*κ*B, occurred in diabetic rat kidneys. Given the diversity of the experimental settings and the uniform pattern of the responses, we speculate that: (1) chronic vascular oxidative stress is a nonspecific (model‐, species‐, organ‐independent) response involving the induction of Nox2 (and Nox5 in guinea‐pigs) and the NF‐*κ*B pathway, and the repression of Nox4 and the Nrf2 pathway; and (2) the systems Nox2‐NF‐*κ*B and Nox4‐Nrf2 regulate each other negatively.

## Introduction

Vascular oxidative stress, characterized by excess vascular production of reactive oxygen species (ROS), endothelial dysfunction, and atheroprone vascular phenotype, and arising from dysregulated redox‐signaling, is a common feature of multiple forms of cardiovascular disease (CVD), including atherosclerosis and diabetic vasculopathies. The atherosclerotic sequel is believed to be as follows (Li and Shah 2004; Burgoyne et al. 2012; Brandes et al. 2014). Cardiovascular risk factors mediate the production of excess vascular superoxide (O_2_^−^). O_2_^−^ initiates cellular redox‐signaling acting: (1) per se or by a product of its dismutation, H_2_O_2_; (2) by O_2_^−^induced inactivation of endothelial nitric oxide (NO, seen as endothelial dysfunction); and/or (3) by peroxynitrite (the reaction product of O_2_^−^ and NO). Ultimately, transcriptional alterations by these ROS and/or reactive nitrogen species (RNS) mediate atherogenic vascular inflammation and remodelling. In line with this scenario: (1) endothelial dysfunction, and its biochemical marker plasma nitrite level (Kleinbongard et al. 2003), correlate with the classical cardiovascular risk‐factor profile and predict the occurrence of myocardial infarction and stroke in humans (Kleinbongard et al. 2006; Giannotti and Landmesser 2007); and (2) endothelial dysfunction and diseased vascular phenotype associating cardiovascular risk factors are accompanied by disorders of redox‐regulated transcription factors, including nuclear factor *κ*B (NF‐*κ*B) activation, and NF‐E2‐related factor‐2 (Nrf2) inactivation, the factors inducing proinflammatory and antioxidant proteins, respectively (Gordon et al. 2011; Tebay et al. 2015; Gimbrone and Garcia‐Cardena 2016). However, the use antioxidants to prevent CVD has proved ineffective in clinical trials (Bjelakovic et al. 2007). The emerging explanation of this paradox is that, while some enzymatic ROS/RNS sources regulate vascular deleterious pathways, other may be vasculoprotective, with their balance determining the actual vascular phenotype (Brandes et al. 2011; Gray et al. 2016). This hypothesis is dealt with further in this study.

The NADPH oxidase (Nox) family of enzymes is a major source of vascular ROS (Stocker and Keaney 2004; Lassegue and Griendling 2010; Brandes et al. 2014). In the cardiovascular system, homologues Nox1, Nox2, Nox4, and Nox5 are expressed (Lassegue and Griendling 2010; Montezano et al. 2011). Nox1/2/5 generate O_2_^−^, and may signal via ROS and/or RNS, and evidence suggests the role for them in mediating endothelial dysfunction and the pro‐atherosclerotic vascular phenotype (Lassegue and Griendling 2010; Bedard et al. 2012; Brandes et al. 2014; Li et al. 2014). In particular, increased vascular expression of Nox1/2/5 has been noted in humans with coronary artery disease or diabetes (Guzik et al. 2008). Human carriers of hereditary Nox2 deficiency are found to have improved endothelial and vascular functions, as compared with healthy subjects (Violi et al. 2013). In addition, knockout studies in atherosclerosis–prone mice confirm important roles for Nox1 and Nox2 in atherogenesis (Barry‐Lane et al. 2001; Judkins et al. 2010; Gray et al. 2013).

Nox4 generates H_2_O_2_ predominantly. It is incapable of inactivating NO and producing peroxynitrite (Takac et al. 2011), and so may engage predominantly in H_2_O_2_‐specific signaling. Nox4 gene overexpression or deletion using murine models has identified: (1) activity that may be Nox4 vasculoprotective (via increased angiogenesis and induction of endothelial NO synthase) (Craige et al. 2011; Ray et al. 2011; Schröder et al. 2012; Peshavariya et al. 2014), reno‐protective (Nlandu et al. 2012), or antiatherogenic (Craige et al. 2015; Schurmann et al. 2015; Di et al. 2016; Gray et al. 2016); (2) Nox4 as an upstream Nrf2 regulator (likely via H_2_O_2_), implicating Nox4 in the conferment of protection via Nox4/Nrf2 pathway (Brewer et al. 2011; Schröder et al. 2012; Smyrnias et al. 2015); and (3) an inverse relationship as regards the vascular expression of Nox2 and Nox4 in mice with either deleted or overexpressed Nox4 (Gray et al. 2016). Likewise, Nox4 or Nox2 deletion have been reported to upregulate the expression of the other in the human pulmonary artery endothelial cells (Pendyala et al. 2009), suggesting that Nox2 and Nox4 may control each other negatively. However, the findings in transgenic mice may not be universally applicable, nor might they be completely relevant to the intact vasculature.

This study was aimed to test whether the mechanism of prolonged vascular oxidative stress, aside from inducing the O_2_^−^ ‐forming Noxs, also involves the suppression of the Nox4/Nrf2 pathway. We have studied two seemingly unrelated models of chronic vascular oxidative stress (season‐ and diabetes‐induced), which have been reported by us previously as inducing Nox2 in the guinea‐pig and rat heart (Konior et al. 2011). Two species were studied because the Nox5 gene is absent from the rat, while being present in guinea‐pigs and human beings (Montezano et al. 2011). We have evaluated cardiac Nox1, Nox2, Nox4, and Nox5 protein expression, and its correlations with: (1) cardiac O_2_^−^ generation and plasma nitrite concentration used as markers of oxidative stress and endothelial dysfunction, respectively; (2) myocardial activity of NF‐*κ*B and Nrf2, and (iii) myocardial expression of several proteins directed by NF‐*κ*B and Nrf2. Some of these associations were also studied in the diabetic rat kidney, as deleterious as well as protective effects of Nox4 have been reported in diabetic kidney disease (Nlandu et al. 2012; Gorin and Wauquier 2015). The results implicate the involvement of repression of the Nox4/Nrf2 pathway in the mechanism underpinning chronic cardiac and renal oxidative stress in vivo.

## Materials and Methods

### Animals

The investigation conforms to the *European Convention for the Protection of Vertebrate Animals used for Experimental and other Scientific Purposes* (Council of Europe No 123, Strasbourg 1985). The local Animal Subject Ethics Committee approved all procedures. Guinea‐pigs of either sex (260–320 g), and male Wistar rats (250–350 g) were maintained under 12‐h light/dark cycle and constant temperature and were studied either in the winter (November‐March 2015 and 2016) or summer (July–August 2015 and 2016). A group of diabetic rats was studied in the winter to avoid complications related to the summer‐associated cardiac O_2_^−^ overproduction (Konior et al. 2011). To induce diabetes, rats were given a single intraperitoneal injection of streptozotocin (STZ, 45 mg·kg^−1^ dissolved in 0.1 mol·L^−1^ citrate buffer) or of citrate buffer, and were sacrificed 7 weeks later. Blood glucose, determined using GlucoDr.auto^TM^ test strips (allmedicus Co., Ltd., Republic of South Korea), amounted to 348 ± 95 and 366 ± 125 mg·dL^−1^ at 1 and 7 weeks after STZ, respectively, versus 89 ± 11 mg·dL^−1^ in the untreated group (Table 1). Prior to sacrifice, the animals were fasted overnight and anesthetized with pentobarbital sodium, 50 mg·kg^−1^ body weight, i.p. The chest was opened, the heart punctured to collect a blood sample, and next mounted on the perfusion apparatus, while kidneys have been washed with saline, freeze‐clamped and stored at −72°C.

**Table 1:** Coronary flow, blood glucose, body weight, ventricle weight/body weight, and kidney/body weight in guinea‐pigs and rats affected by season and STZ‐diabetes

Experimental Group	*N*	Coronary flow (mL·min^−1^)	Blood glucose (mg·dL^−1^)	Body weight (g)	Ventricles wt/body wt (mg·g^−1^)	Kidney wt/body wt (mg·g^−1^)
**Guinea‐pigs**						
Untreated (Winter)	7	11.9 ± 2.1	93 ± 9	361 ± 87	3.25 ± 0.51	
Untreated (Summer)	12	12.2 ± 2.2	89 ± 24	340 ± 101	3.98 ± 0.80^a^	
**Rats**						
Untreated (Winter)	12	11.2 ± 2.6	89 ± 11	314 ± 72	2.83 ± 0.46	3.33 ± 0.06
Untreated (Summer)	9–15	12.6 ± 2.4	97 ± 17	368 ± 21	3.21 ± 0.27^a^	4.17 ± 1.7^a^
STZ‐diabetes (Winter)	12	12.2 ± 4.6	366 ± 125^a^	239 ± 55^a^	2.94 ± 0.35	5.83 ± 0.78^a^

Values are means ± SD of N experiments.

a *P* < 0.05 versus respective Untreated (Winter).

### Isolated heart studies

Unpaced guinea‐pig and rat hearts were Langendorff perfused at 70 mm Hg perfusion pressure with Krebs‐Henseleit buffer (KHB) containing 11 mmol·L^−1^ glucose, and gassed with 95% O_2_ + 5% CO_2_ gas mixture (Konior et al. 2011). The hearts were subjected to 30‐min equilibration perfusion. Then, coronary flow was measured (Transit Time Flowmeter Module, Hugo Sachs Elektronik, March, Germany) while guinea‐pig hearts were evaluated for endothelial function (Section *Endothelium‐Dependent Vasodilatation)*. KHB was then supplemented with cytochrome c and the perfusion was continued over a 20‐min test perfusion during which effluent was collected for cardiac O_2_^−^ generation measurement (Section *Cardiac O*_*2*_^*−*^ *generation*). Finally, the ventricles were weighted, freeze‐clamped, cryopulverized, aliquoted, and stored at −72°C for biochemical analysis.

### Markers of endothelial function

#### Endothelium‐dependent vasodilatation

The vasodilator response to acetylcholine (ACh), serving as a measure of the agonist‐induced endothelium‐dependent vascular function, was studied in isolated guinea‐pig hearts, as described before (Wojtera et al. 2014). Coronary flow was recorded continuously and a 50 *μ*L bolus of 10 *μ*mol·L^‐1^ ACh was injected into the aortic cannula. Next, a 30‐sec sample of the effluent was collected and weighed, with a 30‐sec coronary overflow produced by ACh then being calculated and expressed as a percentage of the basal coronary flow. Data shown are averaged results from two consecutive tests.

#### Plasma nitrite

The hearts were punctured (20 G needle) prior to collection of a 3 mL blood sample into heparinized vacutainer tubes (BD Vacutainer, Lithium Heparine 68 I.U. Becton, Dickinson and Company, UK) as confirmed fairly nitrite contamination‐free in our preliminary studies. The blood was immediately centrifuged at 5000 rpm for 10 min at 4°C. Plasma free of hemolysis (1 mL) was transferred into chilled light protection microtubes, stored at −72°C, and analyzed for nitrite within the next 7 days. Plasma nitrite was measured by the method involving nitrite reduction to NO gas by potassium iodide in glacial acetic acid (a reagent that does not reduce nitrate to NO) as well as the chemiluminescent detection of NO using a Nitric Oxide Analyzer (Sievers Instruments, Inc, Boulder).

### Cardiac O_2_^−^generation

Hearts were perfused with KHB containing 10 *μ*mol·L^‐1^ succinylated ferricytochrome c (Cytochrome c from equine heart, SigmaAldrich) and 600 U mL^‐1^ catalase (Sigma‐Aldrich) (Konior et al. 2011). Perfusate inflowing into the heart and four 1‐min fractions of coronary effluent were collected and their optical density at 550 nm measured. The O_2_^−^ concentration in the effluent was calculated using the molar absorbance coefficient for cytochrome c of 21 mM^−1 ^cm^−1^. Cardiac O_2_^−^ formation was derived from the effluent O_2_^−^ concentration and the coronary flow, and values from the repeat measurements in a given heart were averaged.

### Myocardial NADPH oxidase activity

SOD‐inhibitable NADPH‐mediated O_2_^−^ formation by myocardial lysate served as a measure of combined myocardial activity of O_2_^−^ ‐forming Noxs, but not of H_2_O_2_–forming Nox4. The aliquot of the cryopulverized myocardium was homogenized (TissueLyser LT, Qiagen, Hilden, Germany) in phosphate buffer (50 mmol·L^−1^, pH 7.4) containing 1 mmol·L^−1^ EDTA, 10 mmol·L^−1^ DTT, Halt^TM^ Protease & Phosphatase Single‐Use Inhibitor Cocktail (Thermo Fisher Scientific, Rockford, IL), and 1 mmol·L^−1^ phenylmethylsulfonyl fluoride. The homogenate was centrifuged at 800 g for 10 min at 4°C, and the resulting supernatant used for O_2_^−^ measurement via cytochrome c reduction assay (Konior et al. 2011). Equal protein samples of the supernatant (25 *μ*g, Pierce^TM^ Protein Assay Kit, Thermo Fisher Scientific) were incubated in 1 mL of 50 mmol·L^−1^ phosphate buffer (pH 7.4) containing 20 *μ*mol·L^‐1^ succinylated ferricytochrome c, 3000 U·mL^−1^ catalase, and 100^^ *μ*mol NADPH for 45 min at 37°C, prior to measurement of absorbance at 550 nm (Section *Cardiac O*_*2*_^*−*^ *generation*). All assays were performed in parallel with or without SOD (500 U·mL^−1^).

### Western blot

Protein expression was assessed using the standard Western immunoblot technique. Briefly, ~50 mg samples of the cryopulverized myocardium or kidney cortex were homogenized (TissueLyser LT) in ice‐cold lysis buffer (50 mmol·L^−1^ HEPES, pH 7,4, 150 mmol·L^−1^ NaCl, 10% glycerol, 1% Triton X, 1.5 mmol·L^−1^ MgCl2, 1 mmol·L^−1^ EGTA, 100 mmol·L^−1^ NaF, 10 mmol·L^−1^ Na_4_P_2_O_7_, 1 mmol·L^−1^ NaVO_4_) containing 10 *μ*L·mL^−1^ of Halt^TM^ Protease & Phosphatase Single‐Use Inhibitor Cocktail (Thermo Scientific, Rockford, IL). The homogenate was centrifuged at 13,000 rpm for 10 min at 4°C. Equal protein samples (40–70 *μ*g) of the supernatant were separated on 4–20% Precise^TM^ Protein Gels (Thermo Fisher Scientific) and transferred to nitrocellulose membranes (Amersham^TM^ Protran^TM^ 0.45 *μ*m NC). Blots were probed with monoclonal antibodies for: Nox1 (rabbit, Sigma‐Aldrich Manufacturing, LLC, Saint Luis), Nox2 (gp^91pox^) (rabbit, Sigma‐Aldrich), Nox4 (rabbit, Thermo Fisher Scientific), Nox5 (rabbit, Abcam, Cambridge, UK), eNOS (mouse, Abcam), iNOS (mouse, Abcam), HO‐1 (rabbit, Abcam) or VCAM‐1 (rabbit, Abcam). Equal protein loading was confirmed by *β*‐actin levels (mouse monoclonal antibody, Sigma‐Aldrich). Samples from the animals studied in the winter and the summer, and from the control and the diabetic rats were run on the same blot, to allow straightforward comparison. Protein bands were detected using HRP‐coupled secondary antibodies (polyclonal goat anti‐rabbit or rabbit anti‐mouse, Dako, Glostrup, Denmark) and enhanced chemiluminescent substrate (SuperSignal^®^ West Dura Extended Duration Substrate or SuperSignal^®^ West Pico, Thermo Fisher Scientific). The bands were evaluated by densitometry, and normalized to *β*‐actin values.

### NF‐κB and Nrf2 activation assay

Cardiac and renal NF‐*κ*B and Nrf2 activation was assessed by measuring the p65 (canonical NF‐*κ*B subunit) and Nrf2 contained in tissue nuclear versus cytoplasmic fraction using TransAM^®^ NF‐*κ*B (p65) and TransAM^®^ Nrf2 assay kits (Active Motif). The aliquots of the cryopulverized myocardium or kidney cortex were homogenized (Potter‐Elvehjem Tissue Grinder with Teflon Pestle, 2 cm^3^ size, Wheaton Science Products, Millville, NJ) in ice‐cold 1× Hypotonic Buffer containing protease and phosphatase inhibitors, detergent and dithiothreitol. Tissue nuclear and cytoplasmic fractions, and the nuclear extracts were prepared in line with the manufacturer's instructions. The wells of a respective 96‐well microplate were loaded with 10 *μ*L of the tissue nuclear extract or the tissue cytoplasmic fraction, each containing 20 *μ*g of protein in the NF‐*κ*B (p65) assay and 10 *μ*g of protein in the Nrf2 assay. The absorbance of the wells was read (Microplate Reader, Thermo Lab Multiscan RC, model No 351) versus blank wells containing 10 *μ*L of the Complete Lysis Buffer. Data shown are the results from wells assayed in duplicate.

### Statistical analysis

Data were expressed as mean ± SD if normally distributed. The significance of differences among groups was calculated using one‐way analysis of variance followed by Bonferroni's procedure. Pearson's correlation coefficients were calculated for pairs of continuous variables. A value of *P *<* *0.05 was considered significant.

## Results

### Season‐ and diabetes‐induced O_2_^−^ overproduction and endothelial dysfunction

Untreated guinea‐pigs and rats studied in July–August (summer) versus those studied in November–March (winter) had similar coronary flow, blood glucose and body weight, and respective 22% and 13% increases in the heart ventricles weight/body weight ratio. Rats had also a 25% increase in the kidney weight/body weight ratio, collectively implicating summer‐mediated cardiac and renal hypertrophy. Rats with STZ‐induced diabetes studied in the winter versus untreated rats studied in the winter had hyperglycemia, ~24% lower body weight, similar coronary flow and ventricles weight/body weight ratio, and a 75% increase in the kidney weight/body weight ratio, implicating renal, but not cardiac hypertrophy (Table 1). However, in our previous study we have found STZ‐diabetes to induce cardiac hypertrophy in guinea‐pigs (Konior et al. 2011).

**Figure 1:**
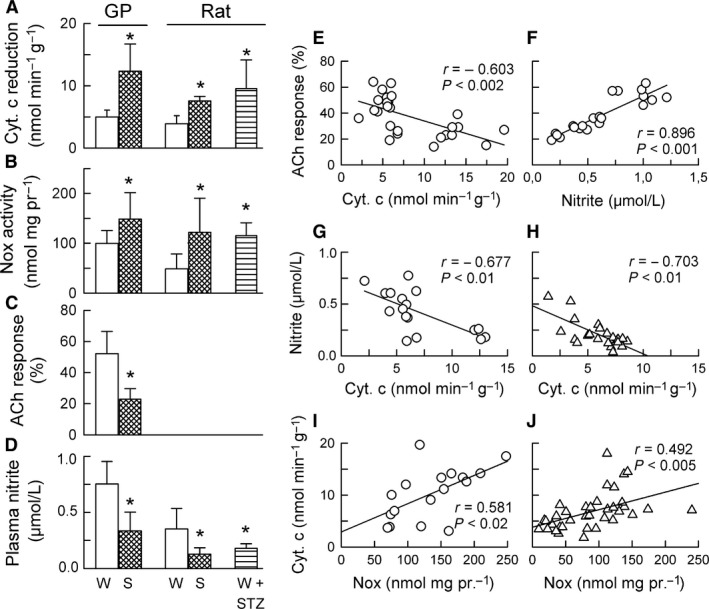
Cardiac O_2_^−^ production (A), myocardial Nox activity (B), coronary flow response to acetylcholine (C), and plasma nitrite concentration (D), as influenced by the season (Winter vs. Summer) in guinea‐pigs (GP) and rats, and by the STZ diabetes induced in rats in the winter (W + STZ). Linear regression analysis of: ACh response versus cardiac O_2_^−^ production (E), and versus plasma nitrite concentration (F) in guinea‐pigs; plasma nitrite concentration versus cardiac O_2_^−^ generation in guinea‐pigs (G, circles) and rats (H, triangles), and cardiac O_2_^−^ generation versus myocardial NADPH oxidase activity in guinea‐pigs (I, circles) and rats (J, tringles). Data plotted were pooled from all guinea‐pig groups and separately from all rat groups studied in A–D. Bars in A–D are means ± SD from 6 to 11 experiments; *P < 0.05 versus respective Winter. In scatterplots, *r* ‐ Pearson correlation coefficient; *P* ‐ significance of the linear relationship.

**Figure 2:**
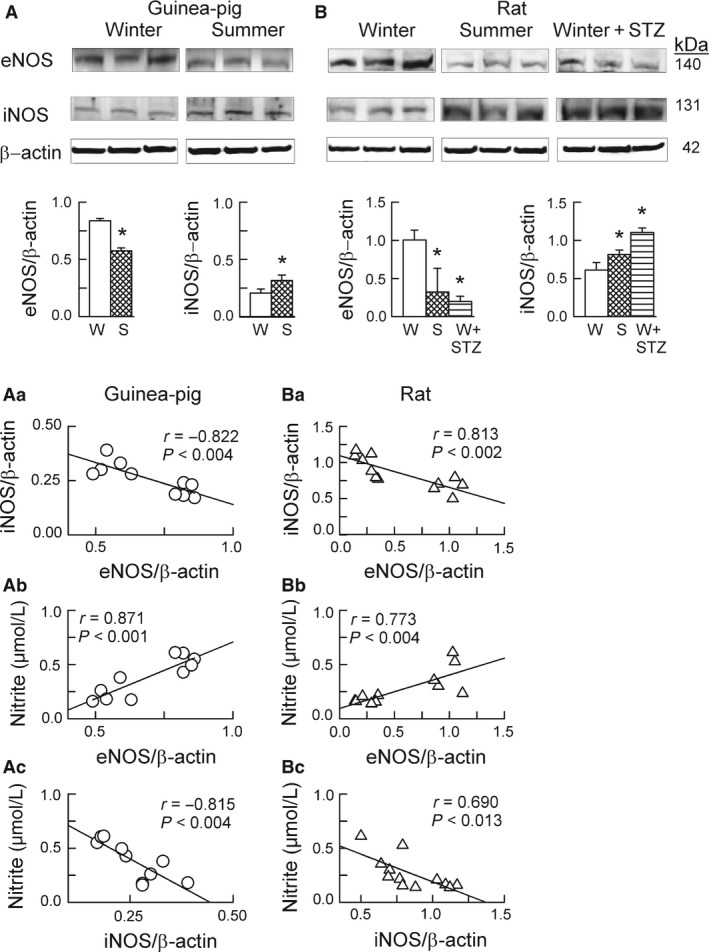
Expression of eNOS and iNOS in guinea‐pig hearts (A) and rat hearts (B), as influenced by the season (Winter vs. Summer) and streptozotocin treatment applied in the winter (W + STZ). Panels A & B depict original Western blots of eNOS and iNOS and of the corresponding *β*‐actin. The bars below represent the protein band intensity evaluated by densitometry and normalized to *β*‐actin. Scatterplots illustrate linear regression analysis of: eNOS and iNOS expression in guinea‐pig hearts (Aa, circles) and rat hearts (Ba, triangles), cardiac eNOS expression and plasma nitrite concentration in guinea‐pigs (Ab) and rats (Bb), and cardiac iNOS expression and plasma nitrite concentration in guinea‐pigs (Ac) and rats (Bc). Data plotted were pooled from all guinea‐pig groups and separately from all rat groups analyzed in A and B. Bars in A–B are means ± SD from 4 to 5 experiments; * *P* < 0.05 versus respective Winter. In scatterplots, r ‐ Pearson correlation coefficient; *P* ‐ significance of the linear relationship.

In line with findings from our earlier work (Konior et al. 2011), guinea‐pig and rat hearts studied in the summer versus in the winter were found to be characterized by significantly increased O_2_^−^ generation (as evidenced by increased outflow of the reduced cytochrome c) (Fig 1A), and increased enzymatic Nox activity (Fig. 1B). Guinea‐pig hearts also had reduced coronary flow response to ACh (Fig. 1C). Summer and diabetes mediated similar increases in O_2_^−^generation and Nox activity in rat hearts (Fig. 1A, B). Furthermore, summer in both species, and diabetes in rats, resulted in: reduced plasma nitrite concentration (Fig. 1 D), reduced myocardial protein level of the endothelial type of NO synthase (eNOS), and increased myocardial protein level of the inducible type of NO synthase (iNOS) (Fig. 2A, B).

**Figure 3:**
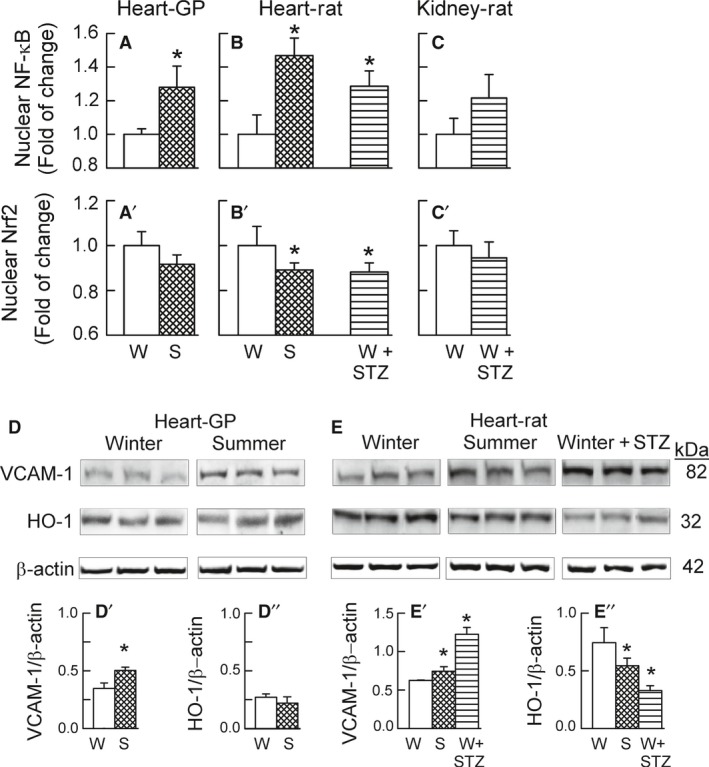
Cardiac and renal activity of NF‐*κ*B and Nrf2 and corresponding cardiac VCAM‐1 and HO‐1 protein levels as influenced by the season (Winter vs. Summer) and streptozotocin treatment applied in the winter (W + STZ). Nuclear accumulation of NF‐*κ*B (p65) (A, B, C) and of Nrf2 (A', B', C') in guinea‐pig hearts (A, A') and rat heart (B, B'), and in rat kidney (C, C') and myocardial expression of VCAM‐1 and HO‐1 (D,E) in guinea‐pig hearts (D, D', D'') and rat hearts (E, E', E''). Bars in A,A'–C,C' are means ± SD from either 5 (A,A' and B,B’) or 4 experiments (C,C’). Panels in D, E depict original Western blots of VCAM‐1 and HO‐1 and of the corresponding *β*‐actin. The bars in D’, D’’ and E', E'' represent the protein band intensity evaluated by densitometry and normalized to *β*‐actin and are means ± SD from 5 experiments; * *P* < 0.05 versus respective Winter.

**Figure 4:**
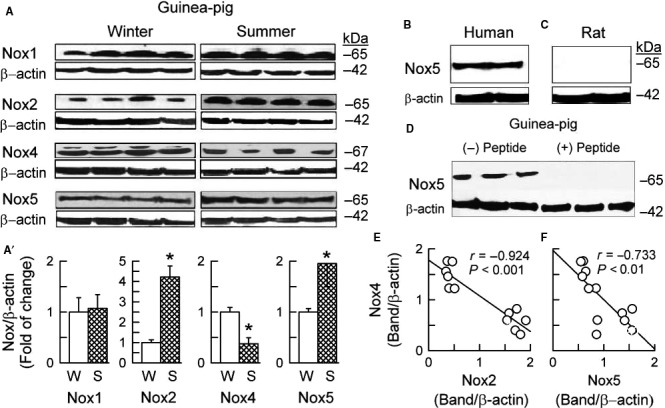
Expression of NADPH oxidase homologues in guinea‐pig heart as influenced by the seasons (Winter vs. Summer). (A) Original Western blots of Nox1, Nox2, Nox4, Nox5, and corresponding blots of *β*‐actin. (A)' The plots represent bands intensity evaluated by densitometry and normalized to *β*‐actin, and are means ± SD of 6–7 experiments.*P < 0.05 versus, respective Winter. The immunoblot with the rabbit anti‐Nox5 polyclonal antibody identified the band at ~65 kDa in human myocardium (B, specimens received by courtesy of Dr. *P*. Leszek, Heart Failure & Transplantation Department, Institute of Cardiology, Warsaw), but not in rat myocardium (C), and the band identified in guinea‐pig myocardium was prevented by Nox5 blocking peptide (the C terminal, Aviva Systems Biology, San Diego, CA) (D). Linear regression analysis of myocardial expression of Nox2 and Nox4 (D) and of Nox5 and Nox4 (E). Plotted are data pooled from all experiments analyzed in (A').

Linear regression analysis of data from Figures 1 and 2 revealed that: (1) in guinea‐pigs, the ACh response was negatively dependent on cardiac O_2_^−^generation (Fig. 1E) and strongly positively dependent on plasma nitrite concentration (Fig. 1F); (2) in guinea‐pigs and rats, plasma nitrite was negatively dependent on cardiac O_2_^−^generation (Fig. 1G and H); (3) in both species, cardiac O_2_^−^generation was weakly positively dependent on the myocardial enzymatic Nox activity (Fig. 1I and J); (4) in both species, the expression of eNOS and iNOS was strongly inversely correlated (Fig. 2Aa and Ba), and (5) in both species, the plasma nitrite concentration was strongly positively correlated with eNOS expression (Fig. 2Ab and Bb), while being negatively correlated with iNOS expression (Fig. 2Ac and Bc).

These data suggest that the endothelial dysfunction in our models, as reflected by an impaired ACh response and/or reduced plasma nitrite, was related to vascular O_2_^−^ overproduction by activated Nox and, at least partially, to impaired endothelial NO production by the downregulated eNOS. The data also imply that plasma nitrite reflects bodily NO generation by eNOS, rather than by iNOS, emphasizing the value of nitrite as a marker of endothelial dysfunction (Kleinbongard et al. 2003, 2006).

### Myocardial and renal NF‐κB and Nrf2 activation

The activity of NF‐*κ*B and Nrf2 was measured in relation to nuclear p65 and Nrf2 accumulation, respectively. Preliminary studies revealed that the measured p65 and Nrf2 concentrations were typically 3 times greater in the cardiac and renal cellular nuclear fractions as opposed to in the respective cytoplasmic fractions, confirming the reproducibility of our tissue subcellular fractionation procedure.

The nuclear accumulation of NF‐*κ*B (p65) was increased significantly (Fig. 3A and B), while that of Nrf2 was reduced slightly and usually significantly (Fig. 3A'and B'), in guinea‐pig and rat hearts harvested in the summer versus in the winter and also in hearts from the diabetic versus untreated rats. The same pattern to changes was evident in kidneys of the diabetic rats though these did not achieve statistical significance (Fig. 3C and C').

In guinea‐pig and rat hearts, the changes in the NF‐*κ*B and Nrf2 activity were paralleled by respective changes in the expression of their target proteins. Thus, the myocardial levels of the NF‐*κ*B‐targeted iNOS (Fig. 2A and B) and of the vascular cell adhesion molecule (VCAM)‐1 (Fig. 3D and E) were increased, while the levels of the Nrf2‐directed heme oxygenase‐1 (HO‐1) (Fig. 3D and E) and eNOS (Fig. 2A and B) were decreased. Previously, we have reported that summer and diabetes resulted in the downregulation of all three isoforms of superoxide dismutase (the other putative Nrf2 downstream targets) in guinea‐pig hearts (Konior et al. 2011), altogether suggesting upregulation of the NF‐*κ*B pathway and downregulation of the Nrf2 pathway, in the studied conditions.

### Myocardial and renal expression of Nox homologues and their associations with cardiac O_2_^−^ generation and NF‐κB and Nrf2 activity

**Figure 5:**
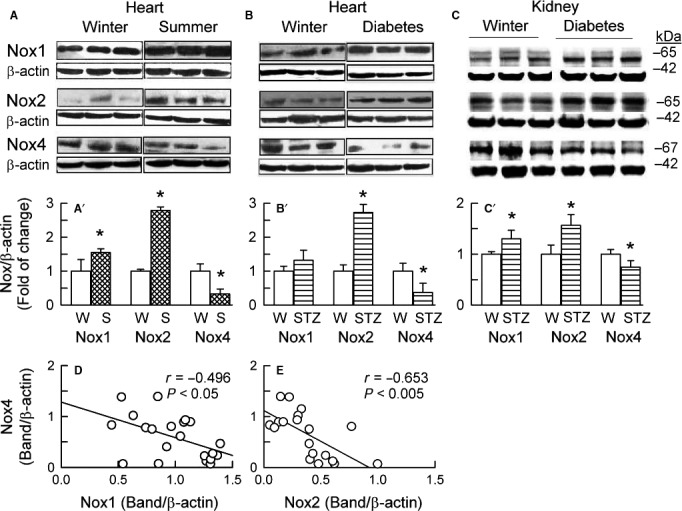
Expression of NADPH oxidase homologues in rat hearts, as influenced by the seasons (Winter vs. Summer) (A) and streptozotocin treatment (Winter vs. Diabetes) (B), as well as in rat kidneys under the influence of diabetes (STZ) (C). Top panels (A, B, C) depict original Western blots of Nox1, Nox2, and Nox4, and corresponding blots of *β*‐actin. The plots below (A', B' C') represent band intensity evaluated by densitometry and normalized to *β*‐actin, the values being means ± SD from 6 to 10 experiments.*P < 0.05 versus respective Winter. Linear regression analysis of myocardial expression of Nox1 and Nox4 (D) and of Nox2 and Nox4 (E). Plotted are data pooled from the experiments in A' and B'.

The summer in guinea‐pig (Fig. 4) and rat heart (Fig. 5A), and diabetes in rat heart (Fig. 5B) and rat kidney (Fig. 5C), all consistently resulted in the many‐fold increases in the Nox2 protein expression and simultaneously in the prominent Nox4 protein downregulation. However, Nox1 and Nox5 behaved differently between the species.

While myocardial levels of Nox1 did not differ between seasons in the guinea‐pig heart (Fig. 4), in rats, summer and diabetes resulted in a small and usually significant myocardial and renal Nox1 upregulation (Fig. 5). As expected, Nox5 protein was not detectable in the rat heart and kidney. Noteworthy, however, is the presence of Nox5 in the guinea‐pig myocardium, as well as its increased expression in summer as opposed to winter (Fig. 4). Indeed, immunoblots using the rabbit anti‐Nox5 (C‐terminal) polyclonal antibody showed a single band at a molecular weight of ~65 kDa in the myocardium of guinea‐pigs (Fig. 4A) or human beings (Fig. 4B, used as a positive control), as well as the lack of a band in the rat myocardium (Fig. 4C). Moreover, the band in the guinea‐pig myocardium was prevented by Nox5‐blocking peptide (C‐terminal) (Fig. 4D), confirming the specificity of the band for Nox5 and the expression of Nox5 in the guinea‐pig myocardium.

With linear regression analysis, it appeared that: (1) Nox4 level correlated inversely with Nox2 and Nox5 levels in the guinea‐pig heart (Fig. 4E and F), and with Nox2 (but not Nox1) levels in the rat heart (Fig. 5D and E); (2) O_2_^−^ generation correlated positively with Nox2 and Nox5 level in the guinea‐pig heart, and with Nox1 and Nox2 levels in the rat heart, while this O_2_^−^ generation was the subject of a strong negative association with Nox4 levels in both the guinea‐pig and rat heart (Table 2); (3) NF‐*κ*B activity correlated positively with Nox2 and Nox5 levels in the guinea‐pig heart and with Nox2 (but not Nox1) levels in the rat heart, while NF‐*κ*B activity and Nox4 level were inversely related in the guinea‐pig and rat heart (Table 2), and (4) Nrf2 activity was positively associated with Nox4 level in the guinea‐pig heart (in the rat heart only a tendency for there be such an association was evident), while being associated negatively with Nox2 and Nox5 levels in the guinea‐pig heart, and with Nox2 level in the rat heart (Table 2).

**Table 2:** Linear regression analysis of cardiac O_2_^−^ generation, and myocardial NF‐*κ*B and Nrf2 activity versus myocardial protein level of NADPH oxidase homologues (Nox)

	Myocardial Nox protein level (Band/*β*‐actin)			
	Nox1	Nox2	Nox4	Nox5
**Guinea‐pig myocardium**				
Cardiac	*r* = 0.129	*r* = 0.911	*r* = −0.823	*r* = 0.856
O_2_^−^ production	*P* = 0.674	*P* < 0.0001	*P* < 0.001	*P* < 0.0002
Myocardial	*r* = 0.084	*r* = 0.919	*r* = −0.894	*r* = 0.834
NF‐*κ*B activity	*P* = 0.828	*P* = 0.004	*P* = 0.007	*P* = 0.02
Myocardial	*r* = 0.152	*r* = −0.652	*r* = 0.777	*r* = −0.626
Nrf2 activity	*P* = 0.744	*P* = 0.035	*P* = 0.039	*P* = 0.031
**Rat myocardium**				
Cardiac	*r* = 0.578	*r* = 0.851	*r* = −0.791	
O_2_^−^ production	*P* < 0.005	*P* < 0.0001	*P* < 0.0001	
Myocardial	*r* = 0.562	*r* = 0.612	*r* = −0.726	
NF‐*κ*B activity	*P* = 0.091	*P* = 0.023	*P* = 0.027	
Myocardial	*r* = 0.099	*r* = −0.747	*r* = 0.592	
Nrf2 activity	*P* = 0.784	*P* = 0.033	*P* = 0.093	

The analysis included data pooled from all studied guinea‐pig heart groups and separately from all rat heart groups.

*r*, Pearson correlation coefficient; *P*, significance of the linear relationship.

## Discussion

The work detailed here demonstrated that the impact of summer in guinea‐pigs and rats, and of diabetes in rats, resulted in: (1) increased cardiac O_2_^−^ production and reduced plasma nitrite concentration, denoting oxidative stress and endothelial dysfunction, respectively; (2) myocardial upregulation of the O_2_^−^forming Noxs and the downregulation of Nox4, and (3) myocardial induction of the NF‐*κ*B pathway and repression of the Nrf2 pathway. The same pattern of changes in Noxs, NF‐*κ*B, and Nrf2 was evident in the diabetic rat kidney. Given the uniform pattern of responses, irrespective of a diversity of experimental settings, we speculate that vascular oxidative stress is a nonspecific (model‐, species‐, and organ‐independent) cellular response involving combined induction of the O_2_^−^forming Noxs and of the proinflammatory NF‐*κ*B pathway and the repression of the vasculoprotective Nox4 and the Nrf2 pathway.

### The diversity of the experimental settings

Two models of chronic vascular oxidative stress, differing in terms of mechanisms, were first studied. One was that induced by the 7‐week STZ‐diabetes (Table 1). The other, associated with the summer season, lasted ca. 2 months, and was of a “physiological” type. We have established before that seasonal oxidative stress occurs in guinea‐pigs and rats between May and September, and that this is a sex‐, temperature‐, light/dark cycle‐, laboratory chow‐, and glycemia‐independent phenomenon (Konior et al. 2011; and Table 1 in this study), leaving still open the question as to its mechanism. Second, we have compared diabetes‐mediated effects in the rat heart versus rat kidney, as two vessel‐reach organs. As Nox4 upregulation has been implicated as the mechanism underlying diabetic nephropathy (Gorin and Wauquier 2015), it was of interest to see whether eventual changes in Nox4 might prove to be the organ‐ versus the experimental context‐related phenomenon? Third, the study involved the rat and the guinea‐pig, two species differing in their cardiovascular expression of Nox5. Homologues Nox1, Nox2, Nox4, and Nox5 are all expressed in humans (Guzik et al. 2000, 2008). Of these, Nox5 is not found in the mouse or rat (Lassegue and Griendling 2010; Montezano et al. 2011). Our studies confirmed the expression of Nox5 protein in the human myocardium, its absence from the rat myocardium, and its presence in the guinea‐pig myocardium (Fig. 4). The latter finding supports the notion that the guinea‐pig is genetically closer to human being than are mice or rats (D'Erchia et al. 1996), also implying that guinea‐pig‐based models of oxidative stress are more relevant to the human (patho) physiology than those involving rodents. Regardless of the diversity of the experimental settings, the pattern of the morphological (Table 1) and biochemical effects (see later) we have encountered was remarkably uniform.

### Differential regulation of Nox1/2/5 and of Nox4

In guinea‐pigs and rats studied in the summer versus in the winter, and in the diabetic rats: (1) the cardiac and renal expression of the O_2_^−^‐forming Noxs (Nox2/Nox5 in the guinea‐pig and Nox2/Nox1 in the rat) was enhanced; (2) the cardiac and renal expression of the H_2_O_2_‐forming Nox4 was depressed, and (3) the cardiac levels of Nox4 appeared strongly inversely correlated with Nox2 (but not Nox1) levels in both species and also with Nox5 levels in the guinea‐pig (Figs. 4,5), overall suggesting that Nox4 and Nox2 (and possibly Nox4 and Nox5) regulate each other's expression. In agreement with this hypothesis, the vascular expression of Nox2 and Nox4 have been found to change reciprocally in mice with Nox2 deletion (You et al. 2013), and with Nox4 deletion or Nox4 overexpression (Gray et al. 2016). In addition, Nox4 or Nox2 deletion have been reported to upregulate the expression of the other in the human pulmonary artery endothelial cells (Nox5 has not been studied) (Pendyala et al. 2009). These data suggest that the cross‐talk between Nox4 and Nox2: (1) is a phenomenon pertinent to transgenic as well as natural conditions; (2) seems to operate universally across species (mouse, rat, guinea‐pig, humans), and organs (heart or kidney), and under different models of oxidative stress (season, diabetes), and (3) may, as such, be of a functional importance although its mechanism remains elusive (see later). Furthermore, in the Nox5‐expressing guinea‐pig (and perhaps in humans), Nox4/Nox5 interactions may also be of a functional importance.

### Noxs versus vascular production of redox mediators

Two observations of this study imply that the increased cardiac expression of the O_2_^−^‐forming Noxs (Nox2/Nox5 in guinea‐pigs and Nox2/Nox1 in rats) resulted in increased cardiac production of O_2_^−^, and probably also of the product of its dismutation, that is, H_2_O_2_. First, cardiac O_2_^−^ ‐generation (as assessed by the increased cyt. c reduction by the cyt. c‐perfused heart) correlated positively with the myocardial enzymatic activity of the O_2_^−^ forming Noxs (Fig. 1I and J) (as assessed by the NADPH‐mediated O_2_^−^ formation in myocardial lysate). Second, myocardial levels of Nox2/5/1 proteins correlated with the whole‐heart O_2_^−^ generation (Table 2), which most likely reflects specifically coronary endothelial O_2_^−^ generation. Indeed, cytochrome c is a large molecule that scarcely penetrates the plasmalemma. This implies that we have measured the intraluminal O_2_^−^: (1) originating from the endothelium, given a short biological O_2_^−^ half‐life, its poor tissue diffusibility, and presumably preserved endothelial integrity in the isolated heart preparation; and more specifically (2) originating from the upregulated endothelial Nox2/5/1, given that they are plasmalemma‐bound enzymes expressed, among others, in the endothelial cells (Lassegue and Griendling 2010), and that Nox is capable of releasing O_2_^−^ intra‐ and extracellularly (Zhang et al. 2007). Collectively, these data support the notion that the increased O_2_^−^ signals we have measured originated from the upregulated endothelial O_2_^−^‐forming Noxs and/or from some other endothelial O_2_^−^ sources induced by the Noxs (Dikalov 2011; Schulz et al. 2014; Wojtera et al. 2014). In line with this, we have reported before that the inhibitors of Nox (apocynin), xanthine oxidase (allopurinol), and NOS (L‐NMMA) have been effective in blocking seasonal O_2_^−^‐overproduction in the guinea‐pig heart (Konior et al. 2011).

While the cardiac and renal H_2_O_2_ production by Nox4 was expected to decline, H_2_O_2_ deriving from the O_2_^−^ dismutation was expected to rise in our models. This made the organ‐based measurement of specifically Nox4‐derived H_2_O_2_ challenging (Nisimoto et al. 2014). Nox4 is constitutively active (Montezano et al. 2011), suggesting that its tissue level may reflect its activity. Nox4 has been identified as an upstream positive regulator of Nrf2 (Brewer et al. 2011; Schröder et al. 2012; Smyrnias et al. 2015). This study demonstrates that summer and diabetes, in parallel with cardiac and renal Nox4 downregulation, resulted in Nrf2 suppression in these organs (Fig. 3, Table 2).

Finally, biological availability of the endothelial NO was compromised in our models of oxidative stress. This was evidenced by the reduction in levels of plasma nitrite in all our models, and by the attenuation of the ACh‐dependent coronary vasodilation in guinea‐pig hearts (the alteration denoting endothelial dysfunction) (Fig. 1). Plasma nitrite has been shown to correlate with endothelial dysfunction in clinical settings (Kleinbongard et al. 2006), and is believed to reflect endothelial NO production by eNOS (Kleinbongard et al. 2003). Indeed, plasma nitrite appeared to correlate positively with myocardial eNOS expression, and negatively with myocardial iNOS expression, in our models (Fig. 2), implicating eNOS, but not iNOS, as a predominant source of plasma nitrite. Theoretically, the mechanism of the reduced bioavailability of the endothelial NO in our models could involve: (1) decreased NO production with downregulated eNOS, a process likely secondary to the downregulation of the Nrf2 pathway (see later); and (2) increased NO inactivation by O_2_^−^ from the overexpressed Nox2/5/1, as suggested by the fact that plasma nitrite appeared inversely related to cardiac O_2_^−^ production in all our models (Fig. 1). Actually, the reaction NO + O_2_^−^ is supposed to result in the increased production of another redox mediator, peroxynitrite (not studied here).

Collectively, the results suggest that the disregulation of the Noxs results in changed vascular production of various putative redox mediators in our models, providing a likely mechanistic link between the disregulation of the Noxs and of the NF‐*κ*B and Nrf2 pathways.

### Noxs versus NF‐κB and Nrf2 pathways

Both NF‐*κ*B and Nrf2 are redox regulated and are known to activate genes coding for inflammation proteins and antioxidant enzymes, respectively (Gordon et al. 2011; Tebay et al. 2015). In the resting state, NF‐*κ*B and Nrf2 are sequestrated in the cytoplasm through association with their repressor proteins called I*κ*B and Keap‐1, respectively. The mechanism of NF‐*κ*B activation relies mainly on ROS‐ and/or RNS‐ (including H_2_O_2_ and peroxynitrite) mediated activation of I*κ*B kinase and the phosphorylation of I*κ*B by the latter (Gordon et al. 2011; Brandes et al. 2014). The activation of Nrf2 involves direct H_2_O_2_‐, NO‐, and/or electrophile‐dependent Keap1 cysteine thiols modification (McMahon et al. 2010; Brandes et al. 2014). Upon stimulation, NF‐*κ*B and Nrf2 are freed from their cytoplasmic repressors and translocate into the nucleus to mediate the transcriptional gene activation.

Summer and diabetes resulted in myocardial NF‐*κ*B activation and Nrf2 inactivation (as assessed by the nuclear accumulation of p65 and Nrf2, respectively), and these changes appeared to correlate with the increased expression of the O_2_^−^ ‐forming Noxs and the decreased expression of Nox4, respectively (Fig. 3, Table 2). Similar relationships were evident in the diabetic rat kidney.

In parallel with NF‐*κ*B activation, myocardial expression of the NF‐*κ*B‐directed proinflammatory iNOS and VCAM‐1 was increased in our models (Fig. 3), implying the activation of the NF‐*κ*B‐mediated cellular‐signaling pathway. Previously, we reported that the summer‐ and diabetes‐mediated upregulation of VCAM‐1, ICAM‐1, and P‐selectin was confined mostly to the coronary endothelium in guinea‐pig and rat hearts (Konior et al. 2011). Altogether, these data suggest that, it was Nox2 in rats, and Nox2 and Nox5 in guinea‐pigs that mediated myocardial activation of the NF‐*κ*B pathway and the proinflammatory coronary phenotype in our models. These data are in agreement with those in the literature showing that insults mediating vascular oxidative stress act via the induction of Nox2 and/or Nox1 and NF‐*κ*B (Lassegue and Griendling 2010; Gordon et al. 2011; Bedard et al. 2012; Brandes et al. 2014; Li et al. 2014). Furthermore, knockout studies in atherosclerosis–prone mice confirmed important roles for Nox1 and Nox2 in atherogenesis (Barry‐Lane et al. 2001; Judkins et al. 2010; Gray et al. 2013), and the above‐mentioned adhesion molecules were shown to participate in both the recruitment of inflammatory cells and the pathogenesis of atherosclerosis (Li and Shah 2004; Stocker and Keaney 2004).

Likewise, Nrf2 inactivation coincided with decreased myocardial expression of Nrf2‐directed HO‐1 (Fig. 3), the enzyme possessing antioxidant and anti‐inflammatory properties in the vascular endothelium (Mason 2016). Previously, we have reported that summer and diabetes resulted in the downregulation of all three isoforms of the antioxidant superoxide dismutase (the other putative Nrf2 downstream targets) in guinea‐pig hearts (Konior et al. 2011). Furthermore, cardiac expression of the vasculoprotective eNOS was suppressed in our models (Fig. 2), the enzyme thought to be regulated by Nox4 and Nrf2 (Craige et al. 2011; Schröder et al. 2012). Thus, it is not only in knockout mice (Brewer et al. 2011; Schröder et al. 2012; Smyrnias et al. 2015), but also in our models that Nox4 is seen to regulate Nrf2 and its downstream target enzymes positively, with the proviso that the putative Nox4/Nrf2 pathway was, in fact, suppressed in our models. The induction of the Nrf2 pathway has been shown to afford vascular and cardiomyocyte protection (Brewer et al. 2011; Schröder et al. 2012; Lopes et al. 2015; Smyrnias et al. 2015),), while its downregulation contributed to redox‐sensitive vascular dysfunction (He et al. 2015; Lopes et al. 2015; Priestley et al. 2016). Thus, both the induction of the Nox2(5)/NF‐*κ*B pathway and the suppression of the Nox4/Nrf2 pathway, as seen in our models, can be viewed as vasculo‐damaging processes (however, see later).

### Physiological relevance

Although it is tempting to interpret our results in terms of the Noxs being upstream regulators of the NF‐*κ*B and Nrf2 pathways, the inverse relationship would be conceivable as well. First, NF‐*κ*B has been found to induce the expression of gp91phox (Nox2), p47phox (a regulatory component of Nox1 and Nox2), and p22phox (a subunit of Nox1, Nox2, and Nox4) in various cell systems (Lassegue and Griendling 2010; Manea et al. 2015). Hence, on the basis of the fact that NF‐*κ*B is redox regulated, a positive Nox2‐NF‐*κ*B feed‐back loop may be considered. Second, Nrf2 induction has been shown to upregulate Nox4, while Nrf2 suppression has conversely been shown to suppress Nox4 and simultaneously to upregulate Nox2 in mouse brain tissue, suggesting that Nrf2 is an upstream positive regulator of Nox4 and a negative regulator of Nox2 (Kovac et al. 2015). Accordingly, the existence of a positive Nox4/Nrf2 feed‐back loop may be considered. Third, NF‐*κ*B and Nrf2 may regulate each other negatively via multiple molecular mechanisms, possibly with no Nox participation (Wardyn et al. 2015). It is conceivable therefore that Noxs and the NF‐*κ*B and Nrf2 pathways function as one multifactorial regulatory system aimed at maintaining cellular redox homeostasis and that the functioning of this system is disturbed at many points in chronic forms of oxidative stress, as seen in this and other studies. Indeed, reduced Nox4 expression has been found in: the VSMCs of stroke‐prone spontaneously hypertensive rats (Lopes et al. 2015), the myocardium of patients with aortic stenosis (Moreno et al. 2013) or diabetes (Tan et al. 2011), and the atherosclerotic plaques of patients with cardiovascular events or diabetes (Gray et al. 2016). However, enhanced Nox4 expression in the aorta of athero‐prone diabetic mice has been found to after 10 weeks of diabetes, albeit reduced again after 20 weeks (Gray et al. 2016). Similar biphasic changes in myocardial Nrf2 (Tan et al. 2011; Bai et al. 2013) and eNOS expression (Nagareddy et al. 2005) have been found in STZ‐induced diabetic rodents. A proposal made in this context (Gray et al. 2016) is that the Nox4/Nrf2 pathway may be overexpressed adaptively to ameliorate oxidative stress in the early stages of various pathologies, while in the more advanced stages, as featured in this study, the tissue antioxidant protection by the Nox4/Nrf2 pathway gets exhausted, adding to the detrimental redox imbalance.

It is puzzling, however, that although the summer‐ and the diabetes‐induced changes proved to be qualitatively similar: (1) those induced by the summer are apparently reversible, suggesting that they fulfill predominantly a regulatory role; and (2) only diabetes has been identified as the cardiovascular risk factor and the inducer of vascular complications. Three alternative hypothesis may be offered to explain this paradox: (1) the seasonal and the diabetic oxidative stress, in fact, differ qualitatively, however, the tests performed in this study have no potential to show this (better tests are needed?); (2) the stresses are qualitatively similar, but it is their different intensity that matters (although it is uncertain if oxidative stress is a gradable vs. an “all‐or‐nothing” type of the response?), and (3) our preferred hypothesis is that as long as oxidative stress is a temporary event (as that induced by the season or 7‐week diabetes) it fulfills a physiological regulatory role (physiologic oxidative stress) and becomes detrimental only when lasts permanently and/or overlaps with already existing vascular disorders (pathologic oxidative stress).”

## Conflict of Interest

No conflicts of interest are declared by the authors.
